# The multifaceted RisA regulon of *Bordetella pertussis*

**DOI:** 10.1038/srep32774

**Published:** 2016-09-13

**Authors:** Loïc Coutte, Ludovic Huot, Rudy Antoine, Stephanie Slupek, Tod J. Merkel, Qing Chen, Scott Stibitz, David Hot, Camille Locht

**Affiliations:** 1 Institut Pasteur de Lille, Center for Infection and Immunity of Lille, Lille, France; 2 Univ. Lille, Lille, France; 3 Centre National de la Recherche Scientifique (CNRS), UMR 8204, Lille, France; 4 Institut National de la Santé et de la Recherche Médicale (Inserm), U1019, Lille, France; 5 Divison of Bacterial, Parasitic and Allergenic Products, Center for Biologics Evaluation and Research, FDA, Silver Spring, MD, USA

## Abstract

The whooping cough agent *Bordetella pertussis* regulates the production of its virulence factors by the BvgA/S system. Phosphorylated BvgA activates the virulence-activated genes (*vag*s) and represses the expression of the virulence-repressed genes (*vrg*s) via the activation of the *bvgR* gene. In modulating conditions, with MgSO_4_, the BvgA/S system is inactive, and the *vrg*s are expressed. Here, we show that the expression of almost all *vrg*s depends on RisA, another transcriptional regulator. We also show that some *vag*s are surprisingly no longer modulated by MgSO_4_ in the *risA*^−^ background. RisA also regulates the expression of other genes, including chemotaxis and flagellar operons, iron-regulated genes, and genes of unknown function, which may or may not be controlled by BvgA/S. We identified RisK as the likely cognate RisA kinase and found that it is important for expression of most, but not all RisA-regulated genes. This was confirmed using the phosphoablative RisAD^60^N and the phosphomimetic RisAD^60^E analogues. Thus the RisA regulon adds a new layer of complexity to *B. pertussis* virulence gene regulation.

Whooping cough or pertussis is a life-threatening respiratory disease and remains one of the major causes of infant mortality despite a global vaccination coverage of >85%. It represents today the most prevalent vaccine-preventable disease in infants^1^, thereby illustrating the shortcomings of current vaccination programs. The development of improved vaccines will certainly benefit from a more thorough understanding of the biology of *Bordetella pertussis*, the main etiologic agent of whooping cough.

*B. pertussis* produces a large array of *bona fide* virulence factors whose production is under the transcriptional control of the two-component system BvgA/S (for review see ref. 2). BvgS is the transmembrane sensor component of the system, which, after a complex cascade of autophosphorylation finally transfers a phosphate group to the cytoplasmic transcriptional regulator BvgA. The BvgA/S system is functional by default, but can be turned off by growth at low temperatures or in the presence of sulphate or nicotinic acid, a process referred to as antigenic or phenotypic modulation^3^,^4^. During the Bvg^+^ or virulence phase, BvgA is phosphorylated and triggers the transcription of the genes coding for adhesins, toxins and other virulence factors. These genes are collectively called virulence-activated genes (*vag*s). By contrast, in the Bvg^−^ or avirulence phase, BvgA is not phosphorylated, and the *vag*s are not expressed. Instead, another set of genes, collectively called virulence-repressed genes (*vrg*s), is expressed in the Bvg^−^ phase^5^.

Two genes were demonstrated to be involved in the regulation of *vrg*s: *bvgR* and *risA*^6^,^7^. RisA is a member of the OmpR family of two-component response regulators and was shown to be required for maximal expression of at least some *vrg*s^7^,^8^. The *risA* gene is adjacent to *risS* coding for the putative sensor kinase of the RisA/S two-component system. Whereas for the closely related animal pathogen *Bordetella bronchiseptica, risA* and *risS* are required for resistance of this organism to oxidative stress and for *in vivo* persistence^9^, *risS* is a pseudogene in the genome of all *B. pertussis* isolates examined so far^8^. These observations suggest that in *B. pertussis* RisA may be an orphan response regulator, that its activity is independent of phosphorylation or that it is phosphorylated by an as yet unidentified kinase distinct from RisS.

In this study we characterized the RisA regulon by whole-genome transcriptomic analysis, identified the likely RisA kinase RisK and examined the role of RisA phosphorylation and of BvgR in RisA activity in *B. pertussis*.

## Results

### Transcriptomic profiling of *B. pertussis* BPSM grown in modulating and non-modulating conditions

**Figure 1:**
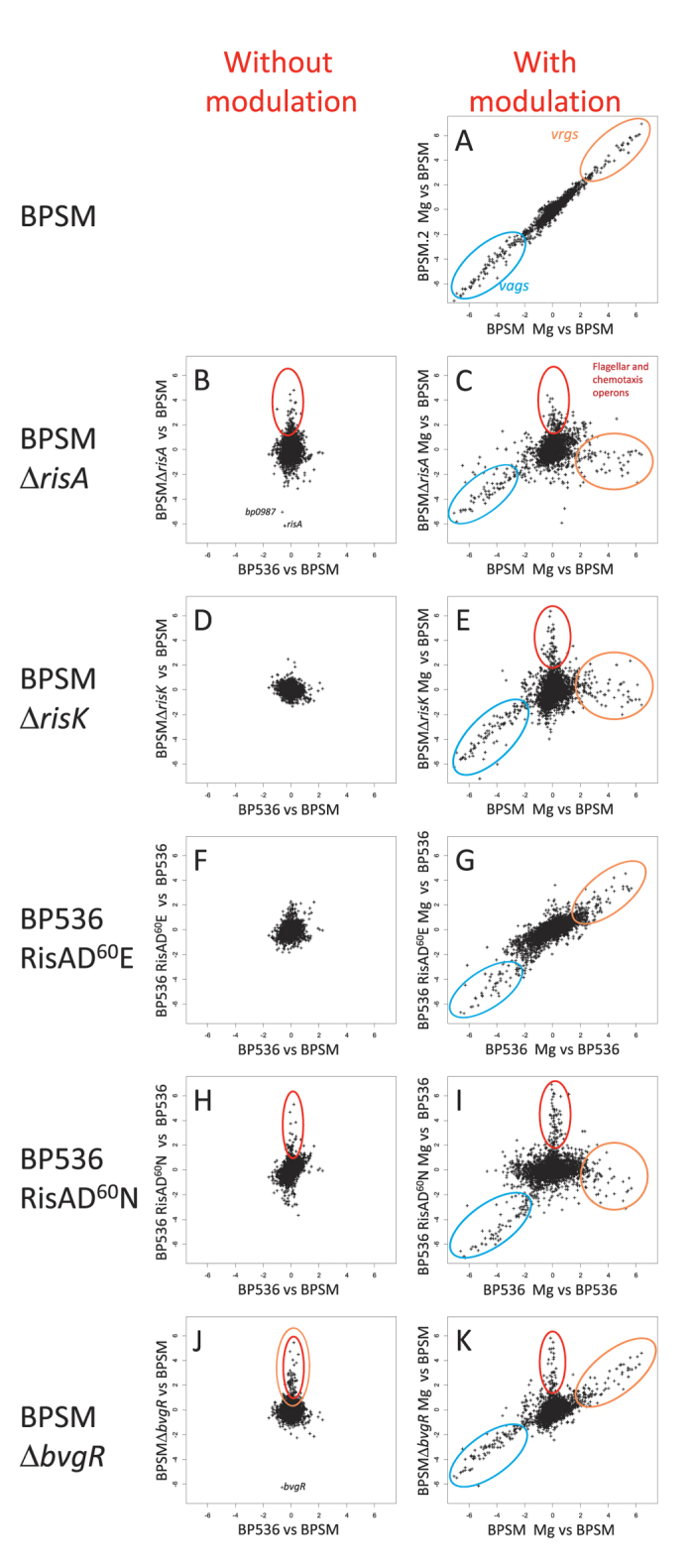
Transcriptome comparisons of different strains in modulating and non-modulating conditions. DNA array gene expression ratios, expressed as log_2_ ratios, between the mutant and parental strains in modulating and non-modulating conditions are depicted in the scatter plots, each point of which represents one gene. In non-modulating conditions, the ratio between a non-modulated mutant strain and its parental strain (*y* axis) is plotted against the ratio between two non-modulated parental strains (x axis). In modulating conditions, the ratio between a modulated mutant strain and its non-modulated parental strain (*y* axis) is plotted against the ratio between the modulated and the non-modulated parental strain (x axis). For the RisAD^60^E and RisAD^60^N mutations the parental strain background is BP536, while for the other strains the parental strain is BPSM. Coloured circles highlight genes of interest; blue for the *vag*s, orange for the *vrg*s and red for genes related to the flagellar and chemotaxis operons. 50 mM MgSO_4_ was used as the modulating condition.

To identify the full set of *vag* and *vrg* genes of *B. pertussis* BPSM, the organism was grown in the presence or absence of 50 mM MgSO_4_ and then subjected to microarray analyses covering 3552 open reading frames. The threshold to identify genes that were differentially transcribed was set at a 4-fold difference in transcript abundance between bacteria grown under modulating and non-modulating growth conditions (Fig. 1A, blue circle and Table S1). The genes more abundantly transcribed in non-modulating conditions than in modulating conditions include the well-known *vag*s, such as the *ptx*/*ptl* operon, *fhaB, bvgA*/*S, bvgR, prn, tcfA, vag8, sphB1, brkA, fim2, fimABC* and some genes involved in siderophore production, whereas the genes more abundantly transcribed in modulating conditions than in non-modulating conditions include the *vrg*s, such as *fim3*, genes encoding the putative capsule, *vrg6* and many genes with unknown function (Fig. 1A, orange circle, see also Fig. S1). These data are in agreement with previously reported transcriptomic profiles of *B. pertussis* grown in modulating versus non-modulating conditions^10^.

### The RisA regulon in non-modulating conditions

**Figure 2:**
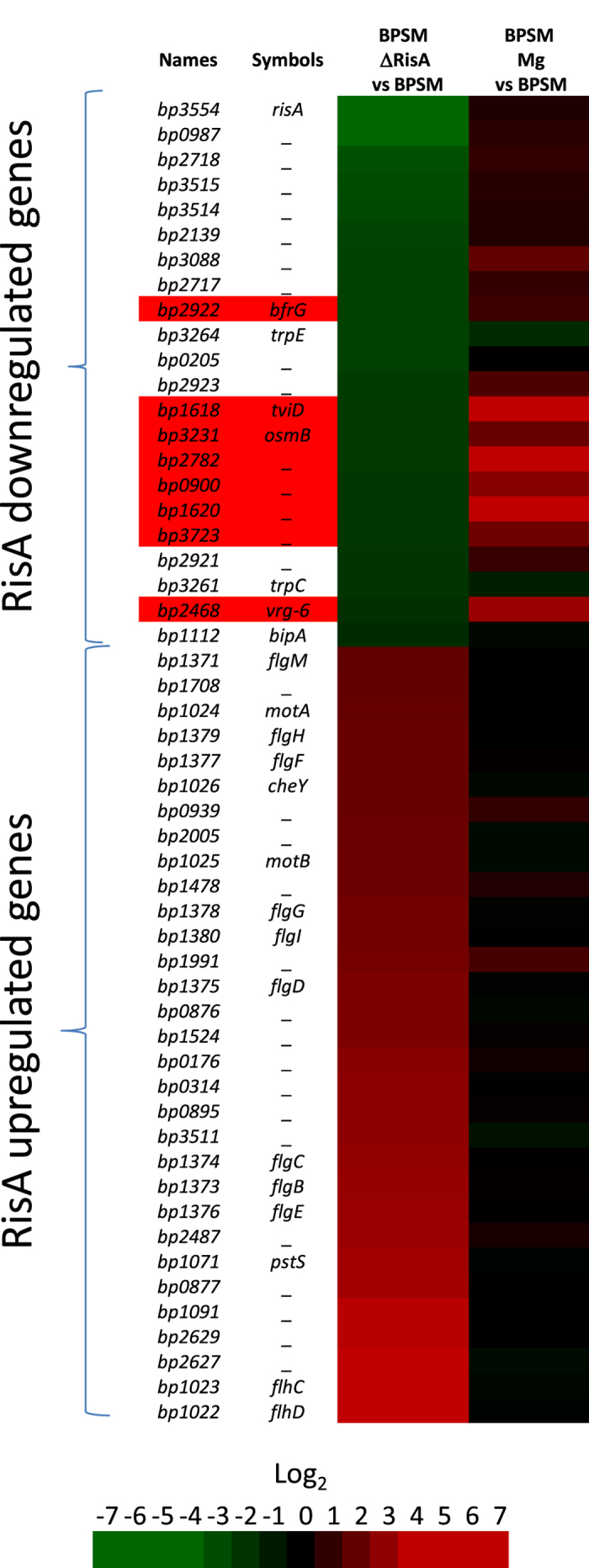
Heat map of RisA-regulated gene expression in non-modulated BPSM. Rows correspond to array probes. The names and symbols in the left column correspond to the Tohama I Sanger Centre annotation. BPSMΔRisA vs BPSM corresponds to the ratios between BPSMΔRisA and BPSM used as reference, both cultivated in non-modulating conditions. BPSM Mg vs BPSM corresponds to the ratios between BPSM cultivated in the presence of 50 mM MgSO_4_ and non-modulated BPSM. The indicated ratios are the means of all the experiments. Data are centred from the first column between genes less transcribed (top) to more transcribed (bottom) in BPSMΔRisA compared to BPSM. Red, increased transcript abundance; green, decreased transcript abundance; black, no significant change in transcript abundance; the level of transcript abundance is defined by the coloured Log_2_ scale shown on the bottom of the figure.

To characterize the RisA regulon, we constructed a *risA*-deficient mutant in the *B. pertussis* BPSM background. The mutant strain, named BPSM∆RisA, carries a 735-bp internal deletion within the *risA* gene. A comparison of the transcriptome of BPSM with that of BPSM∆RisA, both grown in non-modulating conditions, identified 53 genes differentially regulated in BPSM∆RisA compared to BPSM. Among these 53 genes, 22 genes were less abundantly transcribed and 31 genes had higher transcript levels in BPSM∆RisA as compared to BPSM (Figs 1B and 2 and Table S2). Approximately half of the genes with decreased transcript abundance in BPSM∆RisA are *vrg*s (labelled in red in Fig. 2). They include *tviD* (capsular operon), *osmB, vrg-6, bfrG* but also *bipA*, while the remaining genes were not identified as BvgA/S-regulated genes. With the exception of *bp0987*, the gene with the strongest decrease in transcript abundance in BPSM∆RisA (42 fold), all the other genes showed only a 5-fold decrease in transcript abundance (Fig. 1B). Among the 31 genes presenting more transcripts in BPSMΔRisA than in BPSM, none could be identified as BvgA/S-regulated genes. However, many of them belong to the flagellar and chemotaxis operons (Fig. 2). These data suggest that under non-modulating conditions RisA does not regulate *vag* expression but modestly regulates the expression of many *vrg*s in addition to non-BvgA/S regulated genes in *B. pertussis*.

### RisA regulation in modulating conditions

**Figure 3:**
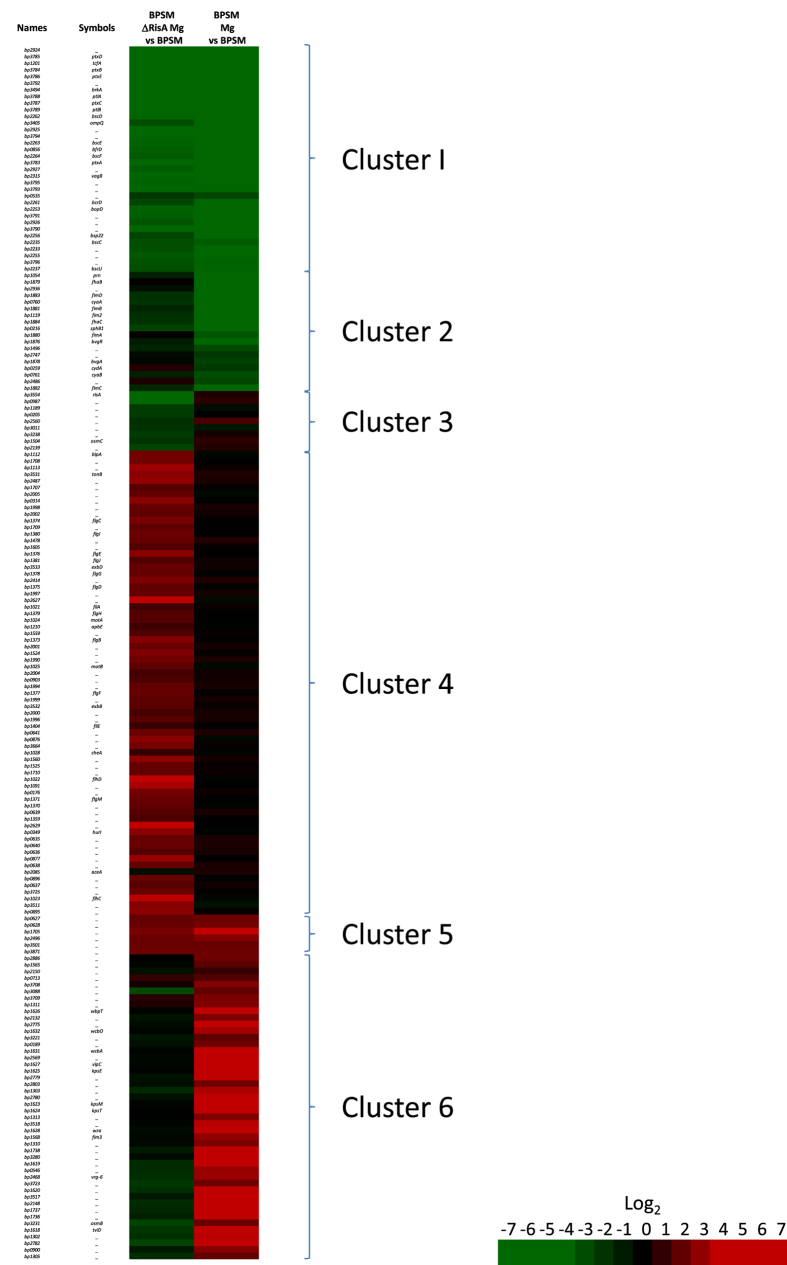
Heat map of RisA-regulated gene expression in modulating conditions. Rows correspond to array probes. The names and symbols in the left column correspond to the Tohama I Sanger Centre annotation. BPSMΔRisA Mg vs BPSM corresponds to the ratios between BPSMΔRisA cultivated in the presence of 50 mM MgSO_4_ and BPSM cultivated in non-modulating conditions. BPSM Mg vs BPSM corresponds to the ratios between BPSM cultivated in the presence of 50 mM MgSO_4_ and BPSM cultivated in non-modulating conditions. The indicated ratios are the means of all the experiments. Red, increased transcript abundance; green, decreased transcript abundance; black, no significant change in transcript abundance; the level of transcript abundance is defined by the coloured Log_2_ scale.

The modest regulation of the *vrg*s by RisA under non-modulating conditions may be enhanced when the bacteria are grown in modulating conditions. Therefore, we compared the transcriptional profiles of BPSMΔRisA and BPSM grown under modulating conditions (in the presence of 50 mM MgSO_4_) to the transcriptional profile of BPSM grown under non-modulating conditions (Fig. 1C). Six different gene clusters were identified (Fig. 3 and Table S3). The first cluster is composed of many *vag*s, which were less transcribed in both modulated BPSM and modulated BPSMΔRisA as compared to non-modulated BPSM, arguing that RisA is not involved in the regulation of most *vag*s, as already proposed by Stenson *et al*.^8^. However, surprisingly, the second cluster is also composed of *vag*s, but they were less transcribed in modulated BPSM than in modulated BPSMΔRisA (Fig. 3). These include the gene coding for pertactin (*prn*), whose transcripts are less abundant in modulated BPSM (fold change of −89.26) than in non-modulated BPSM. In contrast, in modulated BPSMΔRisA, *prn* shows only a 2.34 fold change as compared to non-modulated BPSM (Fig. S2). Other genes that fall in cluster 2 include *fhaB*, genes involved in fimbrial biogenesis and the adenylate cyclase toxin gene. Thus, for some *vag*s the repression by modulation appears to depend on RisA.

The third cluster is composed of genes, such as *osmC* and 7 other genes of unknown function, whose transcripts were less abundant in modulated BPSMΔRisA, but not in modulated BPSM, as compared to non-modulated BPSM. The fourth cluster contains genes that were more abundantly transcribed in modulated BPSMΔRisA than in modulated BPSM (Fig. 3). They comprise genes of the chemotaxis and flagellar operons (Fig. 1C red circle; Fig. S3, labelled in yellow), iron-regulated genes (Fig. S3, labelled in blue) and genes coding for a putative type II secretion system (Fig. S3, labelled in green). Among the genes involved in iron acquisition, some, such as *bfrD*, are regulated by BvgA/S^11^, whereas others, such as *tonB, exbB* and *exbD*, are not. The expression of these genes has been shown to be increased by iron starvation^12^,^13^,^14^.

The fifth cluster is composed of genes that were transcribed at higher levels in both modulated BPSMΔRisA and modulated BPSM as compared to non-modulated BPSM (Fig. 3). Only 6 genes fall in this cluster (*bp0627, bp0628, bp1704, bp2496, bp3501, bp3871*), all of unknown function. Based on their expression in modulated BPSM, these genes would be classified as *vrg*s. However, their expression does not appear to require RisA. The sixth cluster is composed of genes that were much more transcribed in modulated BPSM but not in modulated BPSMΔRisA, compared to non-modulated BPSM (Fig. 3) and contains most of the *vrg*s. Thus, with the exception of the 6 genes in cluster 5, the expression of all the *vrg*s depends on functional RisA.

### Identification of RisK (BP3223) as the putative kinase of RisA

Since RisA is required for the expression of most of the *vrg*s, as well as for a set of genes that do not appear to be modulated by MgSO_4_ (Fig. 3, cluster 4), we further investigated the regulation mechanism of RisA and examined the requirement for its phosphorylation in its activity. To address this issue, we first set out to identify the main kinase involved in RisA phosphorylation. The *B. pertussis* genome contains 17 genes coding for putative two-component system kinases. One of them is *risS*, a gene separated from *risA* by only 4 bp and thus likely co-transcribed with *risA* within the same operon. However, the deletion of *risS* does not perturb the activity of RisA^8^. In addition, *risS* is a pseudogene in *B. pertussis*, in contrast to *B. bronchiseptica*^8^, which makes it unlikely that RisS is the cognate RisA kinase in *B. pertussis*.

**Figure 4:**
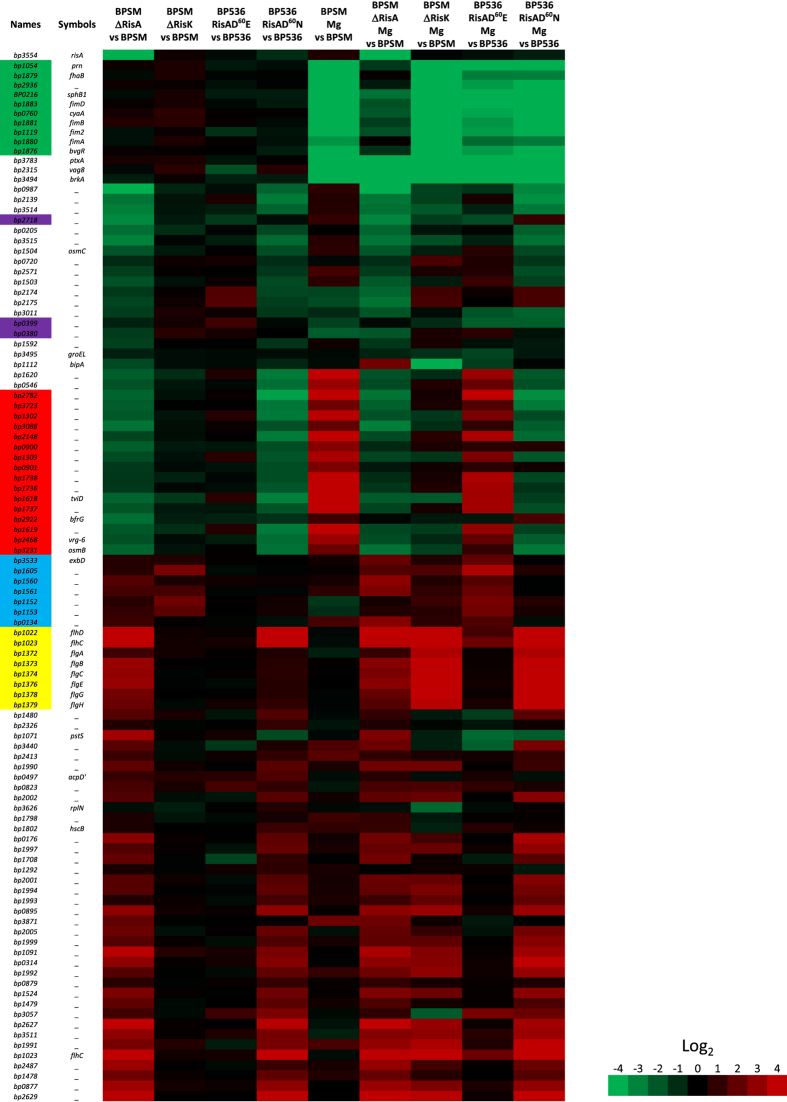
Role of RisA phosphorylation in gene expression in modulated or non-modulated *B. pertussis*. Rows correspond to array probes. The names and symbols in the left column correspond to the Tohama I Sanger Centre annotation. BPSMΔRisA vs BPSM corresponds to the ratios between BPSMΔRisA and BPSM, both cultivated in non-modulating conditions. BPSMΔRisK vs BPSM corresponds to the ratios between BPSMΔRisK and BPSM, both cultivated in non-modulating conditions. BP536 RisAD^60^E vs BP536 corresponds to the ratios between BP536 RisAD^60^E and BP536, both cultivated in non-modulating conditions. BP536 RisAD^60^N vs BP536 corresponds to the ratios between BP536 RisAD^60^N and BP536, both cultivated in non-modulating conditions. BPSM Mg vs BPSM corresponds to the ratios between BPSM cultivated in the presence of 50 mM MgSO_4_ and BPSM cultivated in non-modulating conditions. BPSMΔRisA Mg vs BPSM corresponds to the ratios between BPSMΔRisA cultivated in the presence of 50 mM MgSO_4_ and BPSM cultivated in non-modulating conditions. BPSMΔRisK Mg vs BPSM corresponds to the ratios between BPSMΔRisK cultivated in the presence of 50 mM MgSO_4_ and BPSM cultivated in non-modulating conditions. BP536 RisAD^60^E Mg vs BP536 corresponds to the ratios between BP536 RisAD^60^E cultivated in the presence of 50 mM MgSO_4_ and BP536 cultivated in non-modulating conditions. BP536 RisAD^60^N Mg vs BP536 corresponds to the ratios between BP536 RisAD^60^N cultivated in the presence of 50 mM MgSO_4_ and BP536 cultivated in non-modulating conditions. The indicated ratios are the means of all the experiments. Red, increased transcript abundance; green, decreased transcript abundance; black, no significant change in transcript abundance; the level of transcript abundance is defined by the coloured Log_2_ scale shown on the right.

To search for an alternative RisA kinase, we made use of the concept of co-evolving residue pairs to predict the interacting partner of RisA (http://biohealth.snu.ac.kr/cgi-bin/platcom/tcs/intro.cgi). Among all intact putative *B. pertussis* histidine kinases, RisA was predicted to interact most strongly with the *bp3223* gene product that we therefore propose to call RisK. We deleted the *risK* gene from the BPSM chromosome and analysed the transcriptomic profiles of BPSM∆RisK in modulating and non-modulating conditions. In non-modulating conditions, BPSMΔRisK presented a transcriptomic profile almost identical to that of BPSM and slightly different from that of BPSM∆RisA (Figs 1D and 4 and Table S4). Under modulating conditions, BPSM∆RisK presented a transcriptional profile similar to that of BPSMΔRisA (Figs 1E and 4), indicating that RisA requires the presence of RisK to express its full transcriptional activities. Notable exceptions include some of the *vag*s (Fig. 4, genes in green) and *bipA*, which were more transcribed in modulated BPSMΔRisA than in modulated BPSM∆RisK.

In addition, RisK appears to play a minor role in regulating the expression levels of the RisA-dependent *vrg*s in non-modulating conditions, whereas in modulating conditions, RisK is absolutely required for the upregulation of the expression of these genes (Fig. 4, genes in red). Some of the non-*vrg* RisA-repressed genes (Fig. 3, cluster 4 and Fig. 4, genes in blue) were more abundantly transcribed in modulated BPSMΔRisA than in modulated BPSMΔRisK, whereas others (Fig. 4, genes in yellow) were more transcribed in both modulated BPSMΔRisA and BPSMΔRisK as compared to non-modulated BPSM. Hence, in modulating conditions, some genes require RisK for their RisA-mediated repression, whereas for other genes the involvement of RisK in regulation appears to be dispensable.

To confirm the results obtained by the microarray experiments, quantitative RT-PCR analyses were performed on several selected genes, covering all 6 identified clusters on Fig. 3 in BPSM, BPSMΔRisA and BPSM∆RisK grown in modulating or non-modulating conditions. In all cases the quantitative RT-PCR results confirmed the data obtained by DNA microarray (Fig. S4).

### Transcriptomic profiles of *B. pertussis* RisAD^60^E and RisAD^60^N

To further deepen our understanding of the role of RisA phosphorylation in transcriptional activation, we used *B. pertussis* mutant strains producing phosphomimetic or phosphoablative RisA derivatives. Phosphorylation of two-component response regulators usually occurs at a conserved aspartate residue. Asp-60 of RisA is the most conserved aspartate residue in the RisA/OmpR family of response regulators and is therefore likely to be the site of RisA phosphorylation^8^. RisA was thus genetically replaced by the phosphomimetic RisAD^60^E or the phosphoablative RisAD^60^N analogue, and we compared the transcriptional profiles of the RisAD^60^E- or RisAD^60^N-producing strains to those of the parental strain in modulating and non-modulating conditions.

The phosphomimetic RisAD^60^E mutant presented a transcriptomic profile almost identical to that of its parental strain, both in modulating and in non-modulating conditions, suggesting that the RisAD^60^E protein is fully functional (Figs 1F,G and 4 and Table S4). In contrast, the phosphoablative RisAD^60^N mutant showed many differences with the parental strain, and its transcriptomic profile was similar, but not identical to that of the RisA-deficient mutant (Figs 1H,I and 4), suggesting that the loss of phosphorylation leads to a strong but not complete loss of RisA functions. These observations suggest that some of the RisA-dependent genes do not require phosphorylation of RisA. In non-modulating conditions, genes that are less transcribed in the absence of RisA, but not in the case of its replacement with RisAD^60^N, include genes coding for a putative glycosyl transferase and for a biotin synthase (labelled in purple on Fig. 4). In modulating conditions, all the *vag*s identified in cluster 2 of Fig. 3 (labelled in green in Fig. 4), produced lower levels of transcripts in the RisAD^60^N-producing strain than in BPSMΔRisA. These data suggest that phosphorylated RisA is not required to regulate these genes and confirm that, in addition to modulation, a second level of regulation of these genes is RisA-dependent.

Some genes of the chemotaxis, flagellar and iron acquisition operons (labelled in yellow and blue, respectively, in Fig. 4) appear to be differentially regulated between the RisAD^60^N-producing strain and BPSMΔRisA. Finally, with the exception of the 6 genes of cluster 5 (Fig. 3), the expression of the *vrg*s appears to require phosphorylated RisA, as their transcripts were less abundant in the modulated RisAD^60^N mutant than in the modulated parental strain (Fig. 4, genes highlighted in red). The transcriptome of the RisAD^60^N mutant was very similar to that of BPSM∆RisK, suggesting that phosphorylation of RisA is required for the transcription of most genes belonging to the RisA regulon. However, the transcription of some of them does not require RisA phosphorylation. Furthermore, the effects of the loss of regulation by RisA through the absence of its phosphorylation were enhanced in modulating conditions.

### RisA-mediated regulation in recent clinical *B. pertussis* isolates

**Figure 5:**
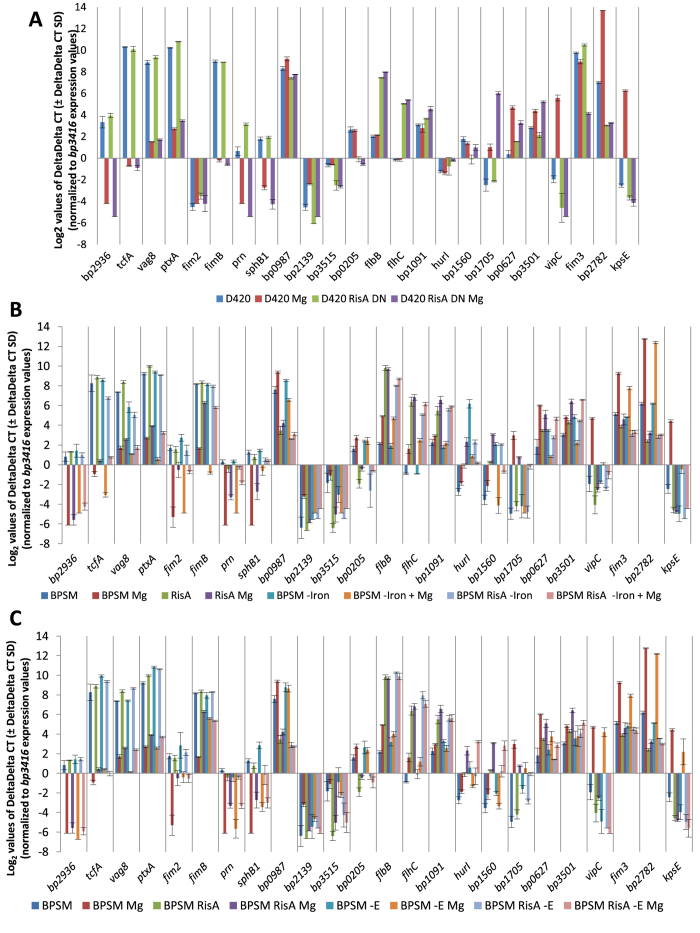
(**A**) Quantitative RT-PCR analysis of genes in D420 and D420 RisAD^60^N. D420 (blue), MgSO_4_ modulated D420 (red), D420 RisAD^60^N (green) and MgSO_4_ modulated D420 RisAD^60^N (violet). (**B**) Quantitative RT-PCR analysis of genes in iron depleted conditions. BPSM (blue), MgSO_4_ modulated BPSM (red), BPSM RisA (green), MgSO_4_ modulated BPSM RisA (violet), iron depleted BPSM (cyan), iron depleted MgSO4 modulated BPSM (orange), iron depleted BPSM RisA (light purple) and iron depleted MgSO4 modulated BPSM RisA (pink). (**C**) Quantitative RT-PCR analysis of genes in glutamate depleted conditions. BPSM (blue), MgSO_4_ modulated BPSM (red), BPSM RisA (green), MgSO_4_ modulated BPSM RisA (violet), glutamate depleted BPSM (cyan), glutamate depleted MgSO4 modulated BPSM (orange), glutamate depleted BPSM RisA (light purple) and glutamate depleted MgSO4 modulated BPSM RisA (pink). The values represent the Log_2_ of mean expression of each gene from mid exponential cultures, normalized to *bp3416* using the 2^ΔΔCt^ method expressed relative to the expression in D420 for (a) or BPSM for (b,c). The error bars represent the ∆∆Ct S.D. 50 mM MgSO_4_ was used as the modulating condition.

Since the above studies were all done with derivatives of the TohamaI laboratory strain, it was important to determine whether a similar role of RisA could be observed in more recent clinical isolates. We therefore attempted to construct *risA* mutants in several clinical isolates, including the highly virulent *B. pertussis* D420 strain^15^. Although several attempts to construct *risA*-deletion derivatives of these clinical strains were unsuccessful, we were able to obtain a D420 derivative in which RisA was replaced by the phosphoablative analogue RisAD^60^N. Using quantitative RT-PCR we compared the transcription of the set of representative genes for each cluster presented in figure S4 in the D420 RisAD^60^N mutant with that of the parent strain, both grown in modulating and in non-modulating conditions. As shown in Fig. 5A, the transcriptional profile of these genes in the D420 background was very similar to the profile seen in the TohamaI derivatives (Fig. S4). Notable exceptions include the *fim2* and *fim3* genes. Since D420 does not produce serotype 2 fimbriae^15^, *fim2* was not expressed in D420, as expected, in contrast to the TohamaI derivatives. The *fim3* gene was highly transcribed in non-modulated D420, non-modulated D420 producing RisAD^60^N and in modulated D420 but was less transcribed in the modulated D420 RisAD^60^N mutant, indicating that *fim3* is not a *vrg* in D420 in contrast to BPSM (see Fig. 3), but has maintained RisA-mediated regulation in modulating conditions.

### RisA-mediated regulation in iron and in glutamate depleted conditions

Since RisA is involved in the regulation of expression of several iron-regulated genes (Fig. S3, labelled in blue), we investigated the role of iron on the expression of the set of representative genes in Fig. 5A by qRT-PCR. BPSM and BPSM∆RisA were therefore grown in iron depleted conditions with or without 50 mM MgSO_4_. As expected, *bp1560* and *hurI* showed a higher transcript abundance in iron depleted conditions than in iron replete conditions, with respective 50.20 and 490.29 fold changes (Fig. 5B). However, the transcriptional profile of the remaining genes in iron depleted conditions was very similar to that seen in iron replete conditions in both BPSM and BPSM∆RisA, arguing that the iron depletion did not modify the regulatory properties of RisA.

Furthermore, it has recently been shown that the depletion of glutamate in the growth medium may also have a major impact on gene regulation in *B. pertussis*^16^. We therefore analyzed by qRT-PCR the transcription of the set of representative genes described above in BPSM and BPSM∆RisA, grown in glutamate depleted conditions with and without 50 mM MgSO_4_. As previously shown^17^, glutamate starvation led to increased transcript abundance of *hurI*, with a 9.96 fold change (Fig. 5C). However, the transcriptional profile of the remaining subset of genes in glutamate-depleted conditions was generally identical to the profile seen in glutamate replete conditions in both BPSM and BPSM∆RisA (Fig. 5C), indicating that, while glutamate depletion has an effect on the transcription of some *B. pertussis* genes, it does not modify the global pattern of regulation of RisA.

### The role of BvgR in RisA-regulated gene expression

**Figure 6:**
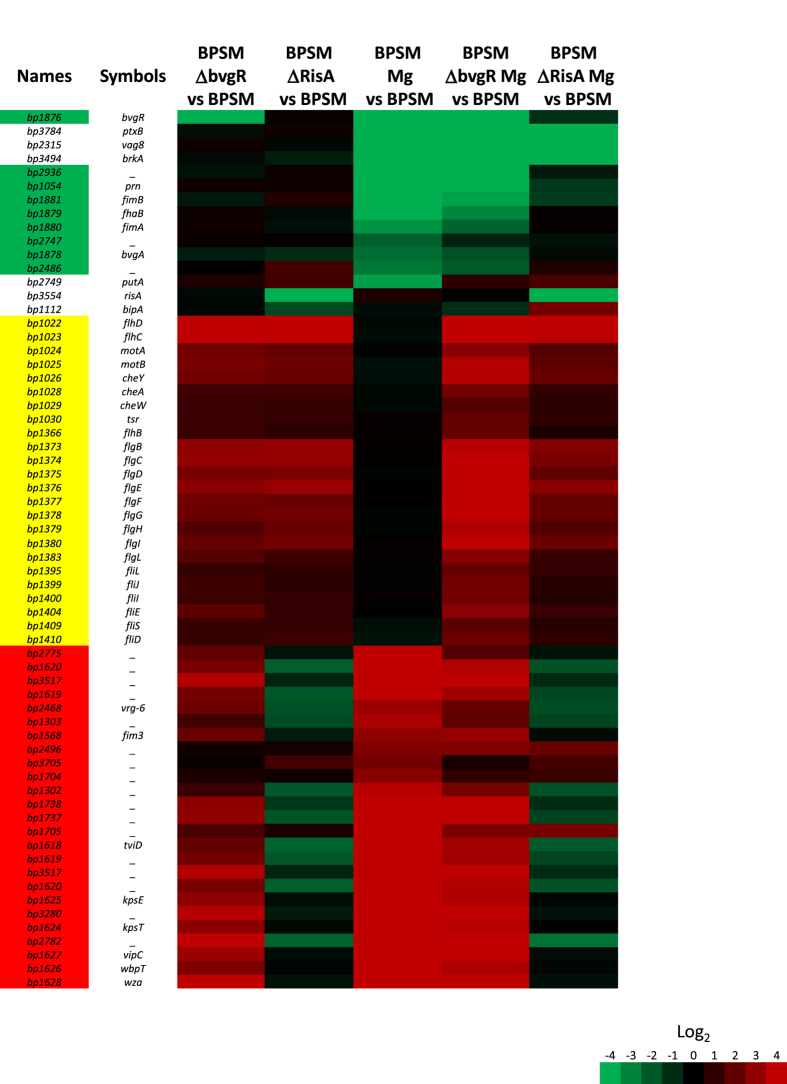
BvgR-regulated gene expression in modulated and non-modulated BPSM and BPSMΔRisA. Rows correspond to array probes. The names and symbols in the left column correspond to the Tohama I Sanger Centre annotation. BPSMΔBvgR vs BPSM corresponds to the ratios between BPSMΔBvgR and BPSM, both cultivated in non-modulating conditions. BPSMΔRisA vs BPSM corresponds to the ratios between BPSMΔRisA and BPSM, both cultivated in non-modulating conditions. BPSM Mg vs BPSM corresponds to the ratios between BPSM cultivated in the presence of 50 mM MgSO_4_ and BPSM cultivated in non-modulating conditions. BPSMΔBvgR Mg vs BPSM corresponds to the ratios between BPSMΔBvgR cultivated in the presence of 50 mM MgSO_4_ and BPSM cultivated in non-modulating conditions. BPSMΔRisA Mg vs BPSM corresponds to the ratios between BPSMΔRisA cultivated in the presence of 50 mM MgSO_4_ and BPSM cultivated in non-modulating conditions. The indicated ratios are the means of all the experiments. Red, increased transcript abundance; green, decreased transcript abundance; black, no significant change in transcript abundance; the level of transcript abundance is defined by the coloured Log_2_ scale shown on the right.

Since expression of the *vrg*s depends on RisA but is also regulated by BvgR and can be modulated by the presence of MgSO_4_, which itself results in reduced *bvgR* expression, we investigated the link between modulation, *bvgR* expression and RisA-dependent transcription. Therefore, the transcriptomic profile of a *bvgR*-deficient strain was compared to that of its parental strain and of the *risA*-deficient strain, all of which were grown under modulating and non-modulating conditions. In non-modulating conditions, the transcripts of most *vrg*s were more abundant in BPSM∆BvgR than in BPSM (Fig. 1J,K, labelled in red in Fig. 6 and Table S5), confirming the role of BvgR in repressing the *vrg*s. As expected, in modulating conditions the *vag*s were less transcribed in both BPSM and BPSM∆BvgR (labelled in green in Fig. 6), whereas the *vrg*s were more transcribed (labelled in red in Fig. 6) as compared to non-modulated BPSM.

Additionally, all the genes related to the flagellar and chemotaxis operons (labelled in yellow in Fig. 6) were also more transcribed in BPSM∆BvgR compared to BPSM in modulating and non-modulating conditions. The comparison of the transcriptional profiles of modulated versus non-modulated BPSM∆BvgR with those of BPSM∆RisA grown in the same conditions indicated that the expression level of most of the chemotaxis and flagellar genes is lower in BPSMΔRisA than in BPSMΔBvgR, especially in modulating conditions. The transcripts of the flagellum-related genes *bp1022* and *bp1023* were more abundant in modulated and non-modulated BPSMΔRisA and BPSM∆BvgR than in non-modulated BPSM. They code for the transcriptional activators FlbB and FlhC, respectively, suggesting that RisA is involved in the repression of these two regulators and therefore may indirectly affect the expression of the remaining genes of the flagellar and chemotaxis operons.

## Discussion

In this study we used microarray analysis to investigate the role of the *risA, risK* and *bvgR* genes in the global *B. pertussis* transcriptomic regulation in both modulating and non-modulating conditions. The vast majority of the genes affected by the *risA, risK* or *bvgR* mutations are all members of the RisA regulon. RisA is a member of the two-component system transcriptional regulator family. However, its putative cognate kinase RisS encoded by the gene located immediately downstream of *risA* is not functional in *B. pertussis* because of a frame shift in *risS*. Instead, we found that the RisK kinase is required for the full regulatory activities of RisA, strongly suggesting that RisK is its cognate kinase. This was confirmed biochemically by some of us using the Phos-Tag technology, showing that the phosphorylation state of RisA is altered in the *risK*-deficient strain (Chen *et al*., submitted).

The transcriptomic profile of BPSM∆RisK, the strain that lacks *risK*, was nearly identical to that of BPSM∆RisA, suggesting that phosphorylation is important for RisA function. However, the expression of some genes was affected by the deletion of *risA* but not by the deletion of *risK*. Since the phosphoablative RisAD60N variant presented the same transcriptional profile as the *risK*-deletion mutant, we conclude that the genes differentially regulated in the *risA* and *risK* mutants are regulated by non-phosphorylated RisA, rather than through phosphorylation via cross-talk with another histidine kinase.

The *risK* gene is co-transcribed with the immediate upstream *bp3222* gene, a member of the *ompR* gene family, located within the same bicistronic operon on the *B. pertussis* chromosome. This gene arrangement is typical for operons that code for two-component systems and suggests that RisK may also be a cognate kinase of the OmpR-like protein and that therefore *risK* deletion may have an impact on the regulatory function of BP3222. However, since the transcriptional profile of BPSM∆RisK was found to be nearly identical to that of the RisAD^60^N mutant, it is likely that RisK exclusively acts on RisA. We found that the transcriptome of a *bp3222*–deficient BPSM derivative was nearly identical to that of BPSM (data not shown). The few genes that were less transcribed in the *bp3222*–deficient strain compared to BPSM, were not thus affected in the *risK*–deficient strain, suggesting either that their expression did not require BP3222 phosphorylation, or that BP3222 is phosphorylated by a kinase different from RisK. In either case, these observations indicate that the transcriptomic profile observed for the *risK* mutant is mediated through the loss of RisA phosphorylation rather than through an effect on the OmpR-like protein.

Many of the genes that are under the control of RisA are *vrg*s. The *risA* deletion affects even the basal level of most *vrg*s in non-modulating conditions, indicating that RisA is required for *vrg* expression even without modulation (Figs 2 and 3). In modulating conditions, the effect of the *risA* deletion is stronger than in non-modulating conditions, as expected. However, the expression of some *vrg*s was not affected by the *risA* deletion in non-modulating conditions. These include *bp0874 (vir-18*) and *fim3*. Thus, RisA may act differentially on a subset of *vrg*s. Alternatively, the basal level of transcription of these genes in non-modulating conditions may be too low to detect a difference between BPSM and BPSM∆RisA. Among the 71 overexpressed genes in modulated versus non-modulated BPSM and therefore identified as *vrg*s, as confirmed by using a ΔBvgA mutant (data not shown), the expression of only 6 was independent of RisA. These 6 RisA-independent *vrg*s may have evolved from the other *vrg*s to become independent of RisA-mediated transcriptional activation or may have additional regulatory systems that override the lack of RisA.

The *vrg*s are also regulated by BvgR, as the presence of this protein represses *vrg* expression. However, binding of BvgR to *vrg* operator sites has not been observed, suggesting that BvgR does not act as a transcriptional repressor. Instead, BvgR contains a conserved EAL sequence found in diguanylate phosphodiesterases that are involved in the degradation of c-di-GMP. Additionally, RisA was shown recently to interact with c-di-GMP (Warfel *et al*., in preparation). Thus, BvgR may participate in the control of intracellular levels of c-di-GMP, a secondary messenger that might regulate the activity of RisA, as has already been shown for other two-component response regulators (for review, see ref. 18).

We found that the expression of the *vrg*s is strongly enhanced in the *bvgR*-deficient strain BPSM∆BvgR, even in non-modulating conditions. This observation suggests that, in order to be fully functional, RisA requires the presence of c-di-GMP, the concentration of which is reduced by the presence of BvgR. Our results also suggest that the expression of some RisA-dependent genes requires phosphorylated RisA but not c-di-GMP, whereas another subset of genes does not require phosphorylated RisA but requires the presence of c-di-GMP.

Interestingly, we have also detected genes that were more abundantly transcribed in BPSM∆RisA than in BPSM in non-modulating conditions. They mainly belong to the flagellar and chemotaxis operons. These genes were not identified as *vrg*s in *B. pertussis*, while they have previously been described as *vrg*s in *B. bronchiseptica*^19^,^20^. In addition, they were also shown to be regulated by c-di-GMP^21^. It remains to be investigated whether they are directly regulated by RisA acting as a repressor, or indirectly involving a regulation intermediate that has yet to be identified. The deletion of *bvgR* resulted in a transcriptomic profile for these genes that is similar to that observed for BPSM∆RisA, with the exception of *flhC* and *flhD*, encoding putative transcriptional regulators. The flagellar genes were also more strongly transcribed in the ∆*bvgR* background compared to the ∆*risA* background in modulating conditions (see Fig. 6), suggesting that c-di-GMP acts at two different levels in the regulation of flagellar gene expression. Hence, in addition to acting with RisA, c-di-GMP may also act on the FlhC and FlhD regulators. A similar mechanism has been shown for the *B. bronchiseptica* flagellum operon^21^, where in modulating conditions or in the absence of BvgR, the basal activity of the FlbB and FlhC regulators is enhanced, resulting in a higher expression of the remaining genes related to the flagellar and chemotaxis operons.

Several genes related to iron acquisition systems were also more transcribed in modulated BPSM∆RisA than in modulated BPSM. It is not known whether RisA acts directly on the expression of these genes or whether it modifies the bacterial perception of the environmental iron concentration. However, not all the genes that were demonstrated to be more transcribed in iron starvation conditions are regulated by RisA. RisA-independent iron-regulated genes include the operon coding for alcaligin biosynthesis (*bp2456-2461*)^12^,^13^,^14^. We also found that iron depletion does not perturb the general pattern of RisA regulation.

Surprisingly, in the RisA-deficient background 18 *vag*s lost their repression mediated by the addition of MgSO_4_ (Fig. 4), whereas they were strongly repressed by the addition of MgSO_4_ in BPSM. In contrast, the expression of all other *vag*s was still repressed in modulated BPSM∆RisA, similarly to modulated BPSM. Several of the former *vag*s, like *prn* and *fhaB*, belong to the class of early *vag*s, while others, like *cyaA*, belong to the class of late *vag*s, suggesting that RisA may act on various kinds of *vag*s involved in the pathogenesis at different times during infection^22^. RisA phosphorylation was not required to suppress the modulatory effect of MgSO_4_ of these *vag*s, while, in contrast, RisA phosphorylation was required for the transcription of all the *vrg*s in modulating conditions.

**Figure 7:**
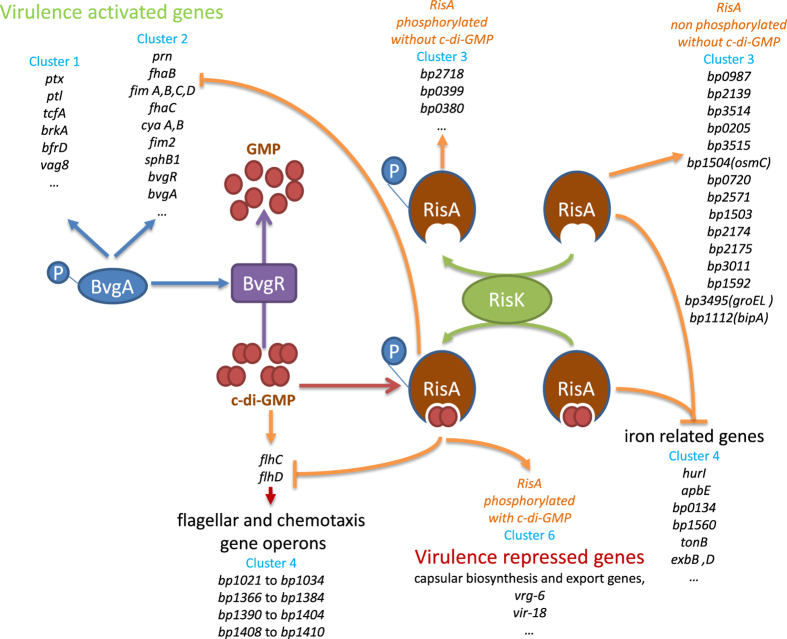
Schematic representation of the RisA regulatory network in *B. pertussis*. Clusters shown in this figure are the same as those identified in Fig. 3. In non-modulating conditions phosphorylated BvgA induces the expression of *vag*s (clusters 1 and 2), including *bvgR*, thereby leading to the degradation of c-di-GMP into GMP. In the absence of c-di-GMP RisK-phosphorylated RisA is able to activate many genes of cluster 3. A subset of cluster 3 genes are also activated by non-phosphorylated RisA in the absence of c-di-GMP. Under modulating conditions, and in the absence of BvgR, binding of c-di-GMP to RisA phosphorylated by RisK leads to the expression of *vrg*s (cluster 6), but also to the repression of flagellar and chemotaxis genes (cluster 4). Unphosphorylated RisA, in the presence or absence of c-di-GMP, inhibits the expression of other cluster 4 genes, including iron-regulated genes. Phosphorylated c-di-GMP-associated RisA is also able to repress the expression of the *vag*s belonging to cluster 2.

In conclusion, the data presented here prompted us to propose a model integrating the roles of *bvgA*/*S, bvgR, risA* and *risK* in the regulation of *B. pertussis* virulence genes (Fig. 7). According to this model, in non-modulating conditions, BvgA is phosphorylated by BvgS and activates the *vag*s, including *bvgR*. Expression of *bvgR* leads to the degradation of intracellular c-di-GMP. RisA is the transcriptional activator of most of the *vrg*s, but in the absence of c-di-GMP RisA is not able to induce the expression of these genes, but can nevertheless activate the basal level of *vrg* expression and repress the expression of the flagellar and chemotaxis genes and of genes involved in iron acquisition. In modulating conditions, BvgS is not active and does not phosphorylate BvgA. Non-phosphorylated BvgA does not activate the expression of *bvgR*. Therefore, the concentration of c-di-GMP increases. The cofactor c-di-GMP binds to phosphorylated and non-phosphorylated RisA participating in the induction or repression of the RisA regulon, including the *vrg*s, the RisA-regulated *vag*s and other genes of unknown function.

Although the role of the RisA regulon in the pathogenesis of pertussis is not yet known, RisA-mediated regulation may perhaps be required to allow the bacteria to adapt to different phases during the infectious cycle. In addition, fine-tuning of adhesin production may contribute to transmission and/or the colonization of a specific niche in the respiratory tract. Furthermore, we have analysed the RisA regulon in standard *in vitro* growth conditions, in iron depleted conditions and in glutamate depleted conditions in the absence or presence of 50 mM MgSO_4_. It remains to be investigated whether the RisA-dependent transcriptome may vary in different growth conditions or during infection. We cannot exclude the possibility that under different growth conditions, additional members of the RisA regulon might be identified.

## Methods

### Construction of *B. pertussis* mutant Strains

The *B. pertussis* strains used in this study were derived from Tohama I derivatives BPSM^23^ or BP536^24^, or from the clinical isolate D420^15^. *B. pertussis* BPSM∆RisA, BPSMΔRisK and BPSMΔBvgR were obtained by homologous recombination using either pSS1129 or pJQ200 mp18 rpsL as allelic exchange vectors^25^,^26^,^27^. The recombinant plasmids were introduced into *B. pertussis* by conjugation via *Escherichia coli* SM10^28^.

BPSMΔRisA carries a 735 bp internal deletion in the *risA* gene (BP3554). It was obtained as follows. Two 800-bp DNA fragments flanking the region to be deleted were obtained by PCR using the BPSM genomic DNA as template and the oligonucleotide pairs 5′-GAATTCGCGGCCACGCCGCCGCCATCCCGCCAGGCC-3′ and 5′-TCTAGAGGCCGGAAATGTAACAGTGA-3′ and 5′-TCTAGACCTAATGGCCCGCCCCGGGC-3′ and 5′-AAGCTTCGCCAGCGGCGTGCACAGGTCGTGTGAAATGCC-3′ as primers. The resulting *Eco*RI-*Xba*I and *Xba*I-*Hin*dIII fragments were then introduced into *Eco*RI-*Hin*dIII-digested pJQ200 mp18 rpsL, yielding pJQ∆RisA. This construct was used for allelic exchange in BPSM, yielding BPSMΔRisA, in which *risS* is located directly downstream of the *risA*/*S* promoter.

BPSMΔRisK carries a 1392-bp internal deletion in the *risK* gene (BP3223). It was obtained as follows. Two 400-bp DNA fragments flanking the region to be deleted were obtained by PCR using the BPSM genomic DNA as template and the oligonucleotide pairs 5′-TATAAAGCTTACGACTACCTCGGCAAGCCCTT-3′ and 5′-TATACTCGAGGCGGAGCAGTTTCATCAGGG-3′ and 5′-TATACTCGAGCCGCTTGCGAAGGCTTGACC-3′ and 5′-TATAGGATCCTGGAGCAATACGGCCCACCT-3′ as primers. The resulting *Hin*dIII-*Xho*I and *Xho*I-*Bam*HI fragments were successively introduced into the *Hin*dIII-*Bam*HI sites of pSS1129, yielding pSS1129 BP3223. This construct was used for allelic exchange in BPSM, yielding BPSMΔRisK. The internal deletion in the *risK* gene led to a truncated RisK protein constituted by the first and last five amino acids of the original RisK protein.

BPSMΔBvgR carries a 804-bp internal deletion in the *bvgR* gene (BP1876). It was obtained as follows. Two 470-bp DNA fragments flanking the region to be deleted were obtained by PCR using the BPSM genomic DNA as template and the oligonucleotide pairs 5′-TATAAAGCTTCAATCCGCGCCATCCAGGTC-3′ and 5′-TATACTCGAGAGCCTCGAAGCTGCTGCGAG-3′ and 5′-TATACTCGAGCGCCGCGAGATGCCGCCCAA-3′ and 5′-TATAGGATCCCGCGCCGGCCACGGACGACG-3′ as primers. The resulting *Hin*dIII-*Xho*I and *Xho*I-*Bam*HI fragments were successively introduced into the *Hin*dIII-*Bam*HI sites of pSS1129, yielding pSS1129 BvgR. This construct was used for allelic exchange in BPSM, yielding BPSMΔBvgR. The internal deletion in the *bvgR* gene led to a truncated BvgR protein constituted by the first 14 and last 9 amino acids of the original BvgR protein.

Strains QC3296 (RisAD^60^N) and BP1942 (RisAD^60^E) were constructed as follows. Plasmid pSS4894 (Chen *et al*., unpublished results) was used as the allelic exchange vector for the construction of pSS5085 (pSS4894::Δ*BP-risA)*, pSS5085.5 (pSS4894::*BP-risA*^D60N^), and pSS5086 (pSS4894::*BP-risA*^D60E^). For each, fragments comprising sequences flanking the mutation were synthesized using PCR amplification with BP536 chromosomal DNA as a template.

For pSS5085, the upstream fragment was amplified with primers 5′-TATAGGTCTCCGGCCGCGGTGGTGAAGGCCACCTTGTC-3′ and 5′-TATAGGTCTCCGGGTTTTGCGTGTTCATGGCCGGAAATGTAACAGTG-3′. The downstream fragment was amplified with primers 5′-TATAGGTCTCAACCCGGATGGCGGCAGTTGACCTAATG-3′ and 5′-TATAGGTCTCGGATCCGATCTGGCCGAGGTCCTCGTCGATG-3′. Both fragments were digested with the type II-S restriction enzyme *Bsa*I to create cohesive ends compatible with *Not*I, *Bam*HI or unique, compatible cohesive ends in the vicinity of the deletion endpoint. The two digested fragments were ligated together with pSS4984 digested with *Not*I and *Bam*HI, transformed, and screened to create pSS5085. For pSS5085.5 the upstream fragment was amplified with primers 5′-TATAGGTCTCCGGCCGCGGTGGTGAAGGCCACCTTGTC-3′ and 5′-TATAGGTCTCCATCAGGTTGAGAACCAGCAGGTCAAAGTG-3′. The downstream fragment was amplified with primers 5-TATAGGTCTCGGATCCGATCTGGCCGAGGTCCTCGTCGATG-3′ and 5′-TATAGGTCTCCTGATGCTGCCGGGCGAGGATGGCCTGTCGATC-3′. For pSS5086 synthesis was similar, with the exception that primer 5′-TATAGGTCTCCATCAGTTCGAGAACCAGCAGGTCAAAGTG-3′ was used, such that the D60E, rather than the D60N, mutation was incorporated.

Transfer of pSS5085 to *B. pertussis* BP536 was accomplished by conjugation following its transformation into the *dap E. coli* strain RHO3, with selection on LB supplemented with gentamicin and DAP. Prior to mating, the recipient BP536 was grown on BG agar plus streptomycin and 50 mM MgSO_4_. Mating was performed by swabbing the *E. coli* donor strain together with the *B. pertussis* recipient strain on BG agar plus MgSO_4_ and DAP. After incubation at 37 °C for 3 hours, bacteria were recovered by swabbing and re-swabbed onto BG agar plus gentamicin (100 μg/ml), streptomycin (50 μg/ml) and 50 mM MgSO_4_ to select for transfer and integration by a single crossover. MgSO_4_ was included to maintain repression of I-*Sce*I synthesis from the pSS4894 vector and the higher level of gentamicin was used to reduce background typically observed under modulating conditions. Discrete colonies arising on these selection plates were then restreaked onto BG agar lacking MgSO_4_ to induce the synthesis of I-*Sce*I enzyme driven by P*ptx*, the subsequent cleavage of the integrated pSS4894 vector, and the stimulation of homologous recombination to repair the resulting double stranded cleavage, resulting in loss of the vector and either incorporation of the mutant allele or a return to the wild-type allele. Colonies that arose on these plates were screened by PCR for incorporation of the deletion allele and also for sensitivity to gentamicin to verify loss of the plasmid vector. In this way strain BP1928 was created. In a similar way, the D60N and D60E mutations were incorporated into the BP536 genetic background using pSS5085.5 and pSS5086 as allelic exchange constructs. However, in these crosses BP1928 was used as the recipient, in order to be able to distinguish the resident deletion allele from the incoming D60 substitution alleles by PCR in the screening step. In this way the strains QC3296 (*risA*^D60N^) and BP1942 (*risA*^D60E^) were created. Strain QC4470 (*B. pertussis* D420, *risA*^D60N^) was constructed as follows QC4470 was created by allelic exchange with the plasmid pQC2266 in *B. pertussis* strain D420. This plasmid was created by cloning a 1 kb gene fragment containing sequences flanking the D60N mutation as well as synonymous changes to introduce a *Sma*I restriction site nearby. The latter was included to facilitate screening of recombinants. The fragment was synthesized as a gBlock (Integrated DNA Technologies, Inc.) with flanking *Bam*HI and *Not*I sites and was cloned, following digestion, into the same sites of pSS4894. The sequence of the fragment is given in Table S6. Transfer of pQC2266 to D420 was accomplished by conjugation following its transformation into the *dap E. coli* RHO3, with selection on LB supplemented with gentamicin and DAP. To maintain modulation in the first stages of this allelic exchange, The recipient D420 was grown on BG agar containing 20 mM MgSO_4_ and mating was performed on BG with 20 mM MgSO_4_ plus DAP. Mating was for 3 hours at 37 °C after which exconjugants were selected after reswabbing on BG agar containing 20 mM MgSO_4_, and gentamicin, but without DAP. Discrete colonies arising on these selection plates were then restreaked onto BG agar lacking MgSO_4_ to allow the cross out of the plasmid vector. Colonies that arose on these plates were screened by sensitivity to gentamicin and PCR of the *risA* region followed by SmaI digestion of the resulting product. The DNA sequence of the *risA* locus in the final strain QC4470 was verified by sequencing the PCR product.

All *B. pertussis* strains were grown on Bordet Gengou agar (BG), in liquid modified Stainer-Scholte medium as described by Locht *et al*.^29^, in liquid iron-depleted Stainer-Scholte medium as described by Alvarez Hayes *et al*.^13^ or in liquid modified Stainer-Scholte medium containing 20% of the standard glutamate concentration, as described by Hanawa *et al*.^16^. The culture media were supplemented with 100 mg/ml streptomycin and 50 mg/ml magnesium sulphate where appropriate.

### Microarray production and analysis

Long oligonucleotide probes were designed on the sequences of the 3552 open reading frames, including all coding sequences, except those of the transposases in IS elements, of *B. pertussis* Tohama I genome using OligoArray v2.1^30^. Oligonucleotides were synthesized by Sigma-Aldrich and spotted on Nexterion AL slides (Schott Nexterion) in 1× SciSpot-AM buffer (Scienion) using a Q-Array II spotter (Genetix). Total RNA was extracted from bacterial pellets harvested from two or three individual cultures for each mutant strain, using TriReagent (Ambion) following manufacturer’s instructions. For each sample, 5 μg of total RNA was reverse transcribed with 400 units of SuperScript III (Invitrogen) in the presence of 100 μM Cy3-dCTP or Cy5-dCTP (GE) and 300 mM of random hexanucleotides (Roche). The labelled cDNA was treated with 1 M NaOH to degrade the RNA and then purified on the Qiaquick PCR purification kit (Qiagen). Hybridization was performed in 40% formamide, 5× Denhardt’s solution, 0.1% SDS, 1 mM Sodium pyrophosphate and 5× SSC during 14–16 h at 52 °C under agitation. Slides were then washed sequentially in 2× SSC/0.2% SDS during 5 min, 0.5× SSC during 10 min, 0.05× SSC during 5 min and 0.01× SSC during 1 min before drying. Hybridized slides were scanned using an Innoscan 700 (Innopsys) microarray scanner and analysed with Mapix v3.1 (Innopsys). For normalisation and differential expression analyses the LIMMA package (Linear Models for Microarray Data)^31^, running under the statistical language R v2.11.1, was used. Statistically significant regulation was identified using moderated t-statistic with empirical Bayes shrinkage of the standard errors^32^. Because of multiple testing, obtained P-values were corrected using the Benjamini & Hochberg method to control false discovery rates^33^. The expression data shown corresponding to each of the 3552 open reading frames were calculated by a means of 2 to 4 individual cultures that were each analysed in 4 technical replicates. All the microarray data are under GEOarchive GSE77754.

### Generation of cDNA

RNA was extracted from bacterial pellets of mid exponential phase cultures grown in Stainer-Scholte medium, using TriReagent (Ambion) following the manufacturer’s instructions. For each sample, 5 μg of total RNA was reverse transcribed with 400 units of SuperScript III (Invitrogen) and 300 mM of random hexanucleotides (Roche). The cDNA products were treated with 1 M NaOH to degrade the RNA and then purified on the Qiaquick PCR purification kit (Qiagen).

### Quantitative real-time polymerase chain reaction

Polymerase chain reaction (PCR) was performed in an Roche LightCycler^®^ 480 Instrument II using 10 μl of 2× Master Mix SybrGreen (Roche), 1 μl of cDNA product (30 ng), 1 μl of 5 μM forward+ reverse primer mix and water to reach a final reaction volume of 20 μl. The primers used are presented in Supplementary Table S6. A 15-min cycle at 95 °C was followed by 40 cycles of 15 s at 95 °C, 8 s at 64 °C and 1 min at 72 °C. At completion of the PCR run, a dissociation curve from 55 °C to 95 °C was run to determine that a single product was generated. The efficiency for each primer pair was determined by serial dilution. The expression of the housekeeping gene *bp3416* was used as reference to normalize the expression of the genes of interest. The experiments were done 3 times with at least 4 technical replicates for each measurement.

## Additional Information

**How to cite this article**: Coutte, L. *et al*. The multifaceted RisA regulon of *Bordetella pertussis.* *Sci. Rep.* **6**, 32774; doi: 10.1038/srep32774 (2016).

## Supplementary Material

Supplementary Information

Supplementary Tables
