# Supplementary material for: The multifaceted RisA regulon of Bordetella pertussis
Source: Sci Rep. 2016 Sep 13;6:32774. doi: 10.1038/srep32774 (PMC5020355; doi:10.1038/srep32774)
Supplement: Supplementary Information [file srep32774-s1.pdf]

The multifaceted RisA regulon of *Bordetella pertussis*

Loïc Coutte[1,2,3,4*], Ludovic Huot[1,2,3,4], Rudy Antoine[1,2,3,4], Stephanie Slupek[1,2,3,4], Tod J. Merkel[5], Qing Chen[5], Scott Stibitz[5], David Hot[1,2,3,4] and Camille Locht[1,2,3,4]

supplementary figures and legends

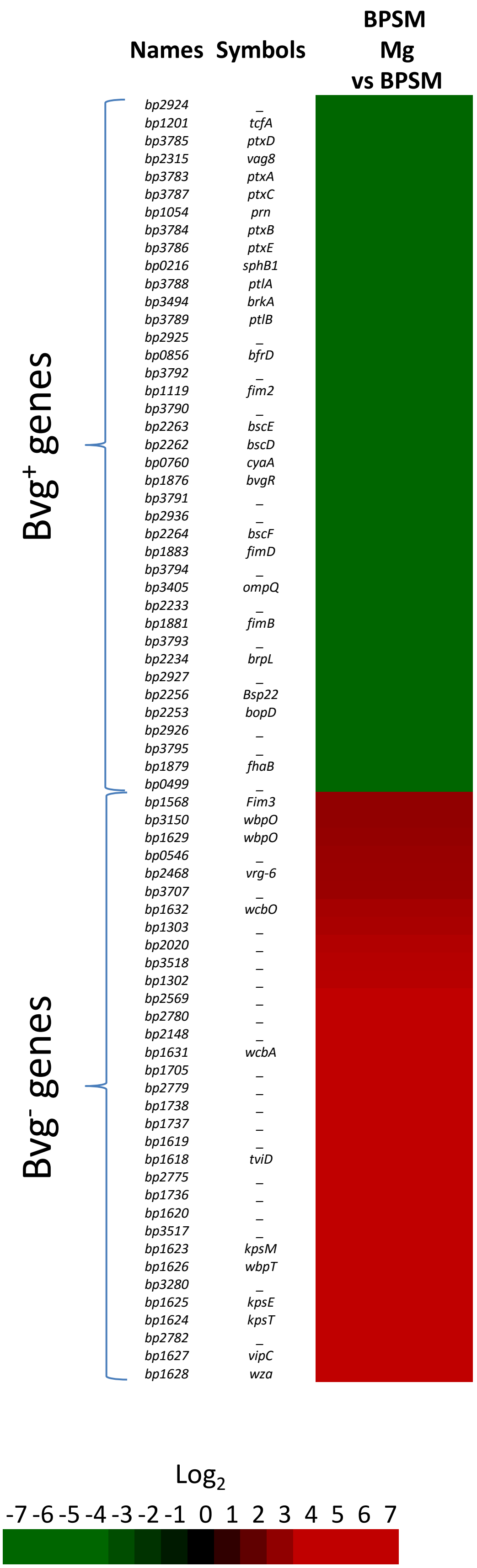

Names
Symbols
BPSM Mg vs BPSM
Bvg+ genes
Bvg- genes
bp2924 _
bp1201 tcfA
bp3785 ptxD
bp2315 vag8
bp3783 ptxA
bp3787 ptxC
bp1054 prn
bp3784 ptxB
bp3786 ptxE
bp0216 sphB1
bp3788 ptlA
bp3494 brkA
bp3789 ptlB
bp2925 _
bp0856 bfrD
bp3792 _
bp1119 fim2
bp3790 _
bp2263 bscE
bp2262 bscD
bp0760 cyaA
bp1876 bvgR
bp3791 _
bp2936 _
bp2264 bscF
bp1883 fimD
bp3794 _
bp3405 ompQ
bp2233 _
bp1881 fimB
bp3793 _
bp2234 brpL
bp2927 _
bp2256 Bsp22
bp2253 bopD
bp2926 _
bp3795 _
bp1879 fhaB
bp0499 _
bp1568 Fim3
bp3150 wbpO
bp1629 wbpO
bp0546 _
bp2468 vrg-6
bp3707 _
bp1632 wcbO
bp1303 _
bp2020 _
bp3518 _
bp1302 _
bp2569 _
bp2780 _
bp2148 _
bp1631 wcbA
bp1705 _
bp2779 _
bp1738 _
bp1737 _
bp1619 _
bp1618 tviD
bp2775 _
bp1736 _
bp1620 _
bp3517 _
bp1623 kpsM
bp1626 wbpT
bp3280 _
bp1625 kpsE
bp1624 kpsT
bp2782 _
bp1627 vipC
bp1628 wza
Log2
-7 -6 -5 -4 -3 -2 -1 0 1 2 3 4 5 6 7

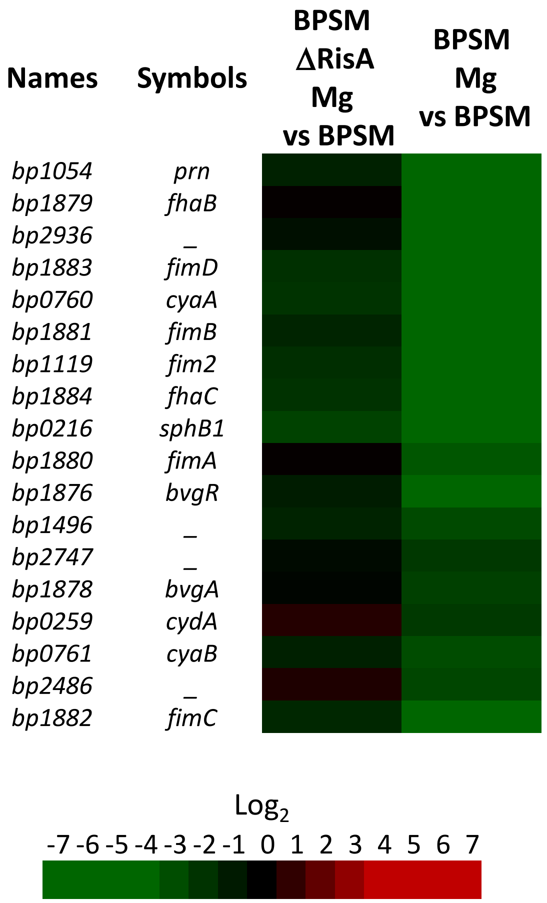
Names
Symbols
BPSM ΔRisA Mg vs BPSM
BPSM Mg vs BPSM
bp1054 prn
bp1879 fhaB
bp2936 _
bp1883 fimD
bp0760 cyaA
bp1881 fimB
bp1119 fim2
bp1884 fhaC
bp0216 sphB1
bp1880 fimA
bp1876 bvgR
bp1496 _
bp2747 _
bp1878 bvgA
bp0259 cydA
bp0761 cyaB
bp2486 _
bp1882 fimC
Log2
-7 -6 -5 -4 -3 -2 -1 0 1 2 3 4 5 6 7

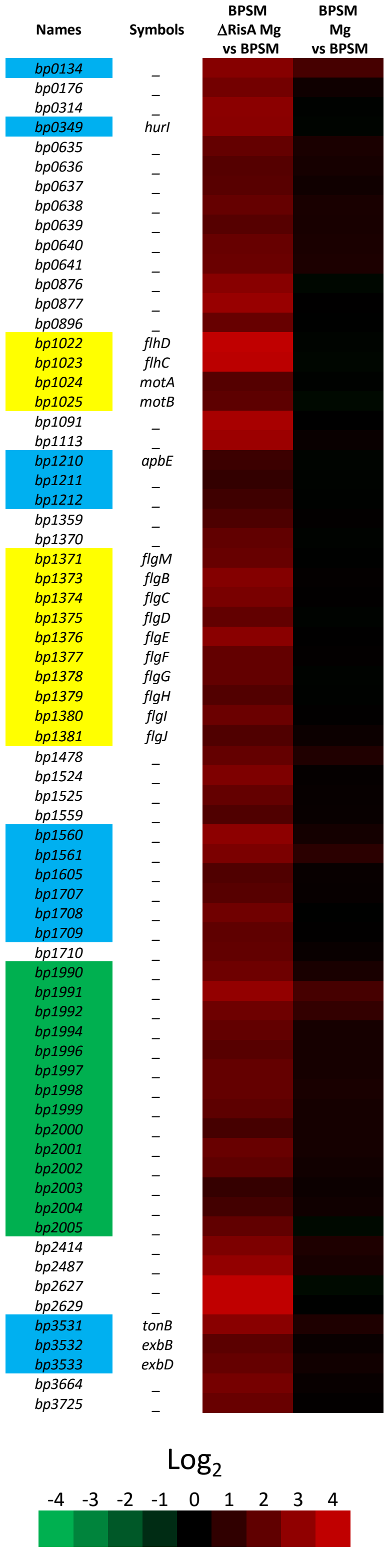

Names
Symbols
BPSM ΔRisA Mg vs BPSM
BPSM Mg vs BPSM
bp0134 _
bp0176 _
bp0314 _
bp0349 hurI
bp0635 _
bp0636 _
bp0637 _
bp0638 _
bp0639 _
bp0640 _
bp0641 _
bp0876 _
bp0877 _
bp0896 _
bp1022 flhD
bp1023 flhC
bp1024 motA
bp1025 motB
bp1091 _
bp1113 _
bp1210 apbE
bp1211 _
bp1212 _
bp1359 _
bp1370 _
bp1371 flgM
bp1373 flgB
bp1374 flgC
bp1375 flgD
bp1376 flgE
bp1377 flgF
bp1378 flgG
bp1379 flgH
bp1380 flgI
bp1381 flgJ
bp1478 _
bp1524 _
bp1525 _
bp1559 _
bp1560 _
bp1561 _
bp1605 _
bp1707 _
bp1708 _
bp1709 _
bp1710 _
bp1990 _
bp1991 _
bp1992 _
bp1994 _
bp1996 _
bp1997 _
bp1998 _
bp1999 _
bp2000 _
bp2001 _
bp2002 _
bp2003 _
bp2004 _
bp2005 _
bp2414 _
bp2487 _
bp2627 _
bp2629 _
bp3531 tonB
bp3532 exbB
bp3533 exbD
bp3664 _
bp3725 _
Log2
-4 -3 -2 -1 0 1 2 3 4

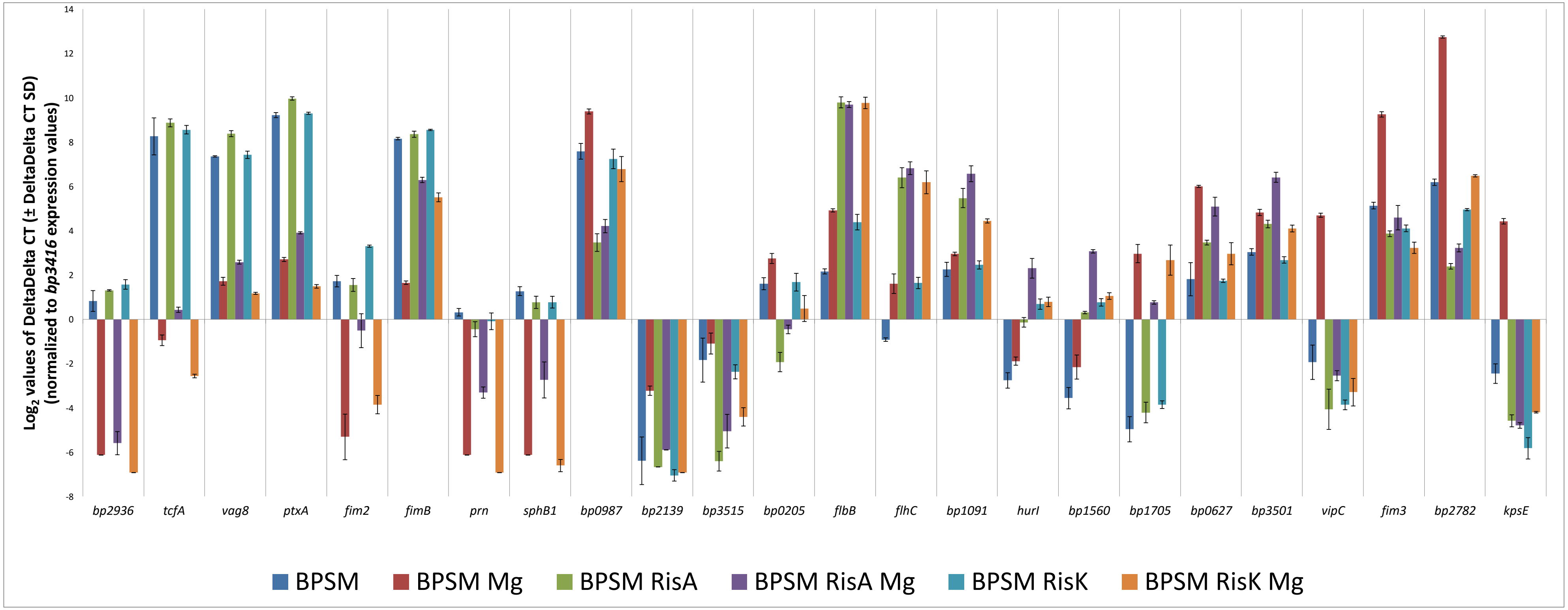

Log2 values of DeltaDelta CT (± DeltaDelta CT SD)
(normalized to bp3416 expression values)
14
12
10
8
6
4
2
0
-2
-4
-6
-8
bp2936
tcfA
vag8
ptxA
fim2
fimB
prn
sphB1
bp0987
bp2139
bp3515
bp0205
flbB
flhC
bp1091
hurI
bp1560
bp1705
bp0627
bp3501
vipC
fim3
bp2782
kpsE
BPSM
BPSM Mg
BPSM RisA
BPSM RisA Mg
BPSM RisK
BPSM RisK Mg

SUPPLEMENTARY FIGURES

Figure S1. Gene expression profiles in modulated and non-modulated BPSM. Rows correspond to array probes. The names and symbols in the left column correspond to the Tohama I Sanger Centre annotation. BPSM Mg vs BPSM corresponds to the ratios between BPSM cultivated in the presence of 50 mM of $MgSO_4$ and BPSM cultivated in non-modulating conditions. The indicated ratios are the means of all the experiments. Only genes modulated by $MgSO_4$ are shown. Red, increased transcript abundance; green, decreased transcript abundance; black, no significant change in transcript abundance; the level of transcript abundance is defined by the coloured Log_2 scale shown on the bottom.

Figure S2. Blow-up of cluster 2 in Fig. 3. Rows correspond to array probes. The names and symbols in the left column correspond to the Tohama I Sanger Centre annotation. BPSMΔRisA Mg vs BPSM corresponds to the ratios between BPSMΔRisA cultivated in the presence of 50 mM of $MgSO_4$ and BPSM cultivated in non-modulating conditions. BPSM Mg vs BPSM corresponds to the ratios between BPSM cultivated in the presence of 50 mM of $MgSO_4$ and BPSM cultivated in non-modulating conditions. The indicated ratios are the means of all the experiments. Green, decreased transcript abundance; black, no significant change in transcript abundance; the level of transcript abundance is defined by the colored Log_2 scale shown on the bottom.

Figure S3. Increased expression of genes in modulated BPSMΔRisA compared to BPSM. Rows correspond to array probes. The names and symbols in the left column correspond to the Tohama I Sanger Centre annotation. BPSMΔRisA Mg vs BPSM corresponds to the ratios between BPSMΔRisA cultivated in the presence of 50 mM of $MgSO_4$ and BPSM cultivated in

non-modulating conditions. BPSM Mg vs BPSM corresponds to the ratios between BPSM cultivated in the presence of 50 mM of $MgSO_4$ and BPSM cultivated in non-modulating conditions. The indicated ratios are the means of all the experiments. Red, increased transcript abundance; green, decreased transcript abundance; black, no significant change in transcript abundance; the level of transcript abundance is defined by the coloured Log_2 scale shown on the bottom.

Figure S4. Quantitative RT-PCR analysis of genes in BPSM, BPSMΔRisA and BPSMΔRisK. BPSM (blue), $MgSO_4$ modulated BPSM (red), BPSMΔRisA (green), $MgSO_4$ modulated BPSMΔRisA (violet), BPSMΔRisK (cyan) and $MgSO_4$ modulated BPSMΔRisK (orange). The values represent the Log_2 of mean expression of each gene from mid exponential cultures, normalized to *bp3416* using the $2^{\Delta\Delta Ct}$ method expressed relative to the expression in BPSM. The error bars represent the ΔΔCt S.D. 50 mM of $MgSO_4$ was used as the modulating condition.
